# Prediction of conditional survival in esophageal cancer in a population-based cohort study

**DOI:** 10.1097/JS9.0000000000000347

**Published:** 2023-04-03

**Authors:** Shao-Hua Xie, Giola Santoni, Matteo Bottai, Eivind Gottlieb-Vedi, Pernilla Lagergren, Jesper Lagergren

**Affiliations:** a School of Public Health and Key Laboratory of Ministry of Education for Gastrointestinal Cancer, Fujian Medical University, Fuzhou, China; b Department of Molecular Medicine and Surgery, Karolinska Institutet, Karolinska University Hospital; c Division of Biostatistics, Institute of Environmental Medicine, Karolinska Institutet, Stockholm, Sweden; d Department of Surgery and Cancer, Faculty of Medicine, Imperial College London; e School of Cancer and Pharmaceutical Sciences, Guy’s Hospital Campus, King’s College London, London, UK

**Keywords:** esophageal neoplasm, mortality, prognosis, prognostic factors

## Abstract

The authors aimed to produce a prediction model for survival at any given date after surgery for esophageal cancer (conditional survival), which has not been done previously.

**Materials and Methods:**

Using joint density functions, the authors developed and validated a prediction model for all-cause and disease-specific mortality after surgery with esophagectomy, for esophageal cancer, conditional on postsurgery survival time. The model performance was assessed by the area under the receiver operating characteristic curve (AUC) and risk calibration, with internal cross-validation. The derivation cohort was a nationwide Swedish population-based cohort of 1027 patients treated in 1987–2010, with follow-up throughout 2016. This validation cohort was another Swedish population-based cohort of 558 patients treated in 2011–2013, with follow-up throughout 2018.

**Results:**

The model predictors were age, sex, education, tumor histology, chemo(radio)therapy, tumor stage, resection margin status, and reoperation. The medians of AUC after internal cross-validation in the derivation cohort were 0.74 (95% CI: 0.69–0.78) for 3-year all-cause mortality, 0.76 (95% CI: 0.72–0.79) for 5-year all-cause mortality, 0.74 (95% CI: 0.70–0.78) for 3-year disease-specific mortality, and 0.75 (95% CI: 0.72–0.79) for 5-year disease-specific mortality. The corresponding AUC values in the validation cohort ranged from 0.71 to 0.73. The model showed good agreement between observed and predicted risks. Complete results for conditional survival any given date between 1 and 5 years of surgery are available from an interactive web-tool: https://sites.google.com/view/pcsec/home.

**Conclusion:**

This novel prediction model provided accurate estimates of conditional survival any time after esophageal cancer surgery. The web-tool may help guide postoperative treatment and follow-up.

## Introduction

HIGHLIGHTS

- We developed a prediction model for conditional survival in esophageal cancer.
- The model had good performance in both internal and external validation.
- The model estimated individuals’ survival any time between 1 and 5 years after surgery.
- A created interactive web-tool may guide postoperative treatment and follow-up.

Esophageal cancer has an overall 5-year survival rate less than 20%^1^,^2^. Surgical resection, often combined with chemo(radio)therapy, is the mainstay of curative treatment^1^,^2^. Tumor stage is the strongest prognostic factor, but several variables influence postsurgery survival, including age, sex, education level, comorbidity, tumor histology, chemo(radio)therapy, resection margin status, and reoperation^3^–^10^.

This study was prompted by a patient asking whether and how his chances of survival had changed after having survived a certain period of time after surgery and depending on his specific prognostic factors. No answer was available in the literature. Yet, prediction of the long-term survival in cancer patients may help plan postoperative treatment and care, and also provide patients and their families with information concerning life expectancy^11^. Predicting conditional survival, that is, the probability of surviving an additional specified period of time given that the patient has already survived for a certain time period after surgery, is a valuable development of conventional baseline prediction models for survival^12^. Conditional survival reflects how the prognosis evolves over time after treatment, and thus provides more accurate estimates of the probability of long-term survival at given time points. A few baseline prediction models have been developed for long-term postoperative survival in esophageal cancer patients, but not estimated conditional survival^13^–^17^.

We aimed to: develop and evaluate a prediction model to estimate conditional survival after surgery for esophageal cancer; externally validate the accuracy of this model; and construct a web-based interactive calculator for prediction of the remaining survival based on model variables at any given date between 1 and 5 years of surgery.

## Materials and methods

### Design

We developed and validated a prediction model of conditional survival in patients having had curative surgery for esophageal cancer and survived greater than or equal to 1 year after surgery using two nationwide Swedish population-based cohorts.

The derivation cohort has been described in detail elsewhere^3^,^6^,^17^–^21^. It consisted of patients (98% participation rate) treated in 1987–2010. Patients were followed until death or end of study (31 December 2016), whichever occurred first, allowing 5-year follow-up for all.

The external validation cohort included esophageal cancer patients who had undergone surgery in 2011–2013. Patients were followed up until death or end of the study (31 December 2018), whichever occurred first, again allowing 5 years follow-up for all.

In both cohort, comprehensive data were retrieved from Medical Records and National Swedish Health Data Registries, that is, registries for cancer, patients, death, prescribed drugs, and education. The work has been reported in line with the STROCSS criteria^22^, Supplemental Digital Content 1, http://links.lww.com/JS9/A224.

### Outcomes

The main outcomes were all-cause mortality and disease-specific mortality any time within 5 years of surgery. Mortality data were retrieved from the National Swedish Cause of Death Registry, which has 100% completeness in the assessment of the date of death (all-cause mortality) and greater than 96% completeness for cause of death (disease-specific mortality).

### Candidate predictors

Nine candidate predictor variables were considered: age (continuous); sex (male or female); education (≤12 or >12 years of formal education); comorbidity (Charlson comorbidity index score, categorized into 0, 1, or greater than or equal to 2, excluding the esophageal cancer diagnosis)^23^; tumor histology (adenocarcinoma or squamous cell carcinoma); neoadjuvant chemo(radio)therapy (yes or no); pathological tumor stage (0–I, II, III, or IV); resection margin status [tumor-free (R0 resection) or tumor involvement (R1/R2)]; and (9) reoperation within 30 days of surgery (yes or no).

### Model development

We created our own novel model to assess probability of survival. The survival probability depends on the predictors and the postoperative day the probability was calculated. For any given patient, the survival probability increases over time, approaching the value one as the time after surgery increases. The conditional survival was modeled by maximizing the likelihood function $l\left(\alpha^{T},\beta^{T}\mathrm{;t},d,x^{T}\right)=\prod_{i=1}^{n}f_{1}\left(x_{i}^{T}\right)f_{2}\left(d_{i}|x_{i}^{T};\alpha^{T}\right)f_{3}\left(t_{i}|d_{i},x_{i}^{T};\beta^{T}\right)$ with the unknown parameters *α* and *β*to be estimated. In the above equation, x was the vector of patients’ predictors, *t* was the time the patients experienced an event, and *d* denoted the type of event the patient experienced, where censoring was defined by *d* = 0, death due to esophageal cancer by *d* = 1, and death due to any other cause by *d* = 2.

Function $f_{1}\left(x_{i}^{T}\right)$ was removed from the maximization of the likelihood function because it did not depend on the unknown parameters α and β . Function $f_{2}$ represented the conditional probability of dying from a competing event. Because there were two competing events (all-cause and disease-specific mortality), function $f_{2}$ was set equal to a logistic function, that is, for competing risk of death due to esophageal cancer $f_{2}\left(d_{i}|x_{i}^{T};\alpha^{T}\right)={\text{logit}}^{-1}\left(x_{i}^{T}\alpha \right)$ .

Function $f_{3}$ represented the conditional density function of time to event defined as $f_{3}\left(t_{i}|d_{i},x_{i}^{T};\beta^{T}\right)={f_{4}\left(t_{i}|d_{i},x_{i}^{T};\beta^{T}\right)}^{I\left(d_{i}\neq 0\right)}{f_{5}\left(t_{i}|x_{i}^{T};\beta^{T}\right)}^{I\left(d_{i}=0\right)}$ , that is the product of the conditional probability of dying between time $t_{i}$ and time $t_{i}+1$ , i.e. $f_{4}\left(t_{i}|d_{i},x_{i}^{T};\beta^{T}\right)=S_{4}\left(t_{i}|d_{i},x_{i}^{T};\beta^{T}\right)-S_{4}\left(t_{i}+1|d_{i},x_{i}^{T};\beta^{T}\right)$ , where $S_{4}$ is the parametric survival function; and the conditional probability of being alive at time $t_{i}$ , that is $f_{5}\left(t_{i}|x_{i}^{T};\beta^{T}\right)=\sum_{j=1}^{2}S_{4}\left(t_{i}|d_{i}=j,x_{i}^{T};\beta^{T}\right)f_{2}\left(d_{i}=j|x_{i}^{T};\alpha^{T}\right)$ , with j indicating the type of competing event.

Three distributions were tested to select the functional form of function $S_{4}$ : log-logistic, Weibull, and Gompertz distribution. The final model survival function followed a log-logistic distribution: $S_{4}\left(t_{i}|d_{i},x_{i}^{T};\beta^{T}\right)=1-1/\left(1+\mathrm{ex}p^{-v\left(t_{i}|d_{i},x_{i}^{T};\beta^{T}\right)}\right)$ and was selected because this distribution has a closed form solution and hence it was more stable than the Weibull distribution and Gompertz distribution and had an Akaike information criterion value similar to that of the other two distributions. Goodness of fit of the model without candidate predictors was tested by plotting the Kaplan–Meier curve versus the survival function $f_{5}$ .

The function *v* was equal to $v\left(t_{i}|d_{i},x_{i}^{T};\beta^{T}\right)=\left(d_{i}=1\right)\gamma +{\times x}_{i}^{T}\eta +\log{\left(t_{i}\right)}^{T}\phi +\left(d_{i}=1\right)\log{\left(t_{i}\right)}^{T}\rho .$ Splines of the term $\log\left(t_{i}\right)$ did not improve the model. The final model for the likelihood function to maximize was equal to:


$$l\left(\alpha^{T},\beta^{T}\mathrm{;t},d,x^{T}\right)=\prod_{i=1}^{2}\left[{d_{i}-1+{\left(-1\right)}^{d_{i}-1}\mathrm{logit}}^{-1}\left(x_{i}^{T}\alpha \right)\right]{\left[1-1/\left(1+\mathrm{ex}p^{-v\left(t_{i}|d_{i},x_{i}^{T};\beta^{T}\right)}\right)-1-1/\left(1+\mathrm{ex}p^{-v\left(t_{i}+1|d_{i},x_{i}^{T};\beta^{T}\right)}\right)\right]}^{I\left(d_{i}\neq 0\right)}{\left[1-1/\left(1+\mathrm{ex}p^{-v\left(t_{i}|d_{i}=1,x_{i}^{T};\beta^{T}\right)}\right){\mathrm{logit}}^{-1}\left(x_{i}^{T}\alpha \right)+1-1/\left(1+\mathrm{ex}p^{-v\left(t_{i}|d_{i}=2,x_{i}^{T};\beta^{T}\right)}\right)\left(1-{\mathrm{logit}}^{-1}\left(x_{i}^{T}\alpha \right)\right)\right]}^{I\left(d_{i}=0\right)}$$

Parameters α , η , ϕ , γ , and ρ were functions of the candidate predictors. Predictors remaining in the final model were selected using the following strategy. Sex and age were selected a priori and were kept only in parameters η and α . The other variables were tested separately in each parameter in this order, first in η , then in α , ϕ , γ , and lastly in ρ . A variable was left in the model if the *P* value was less than 0.05. For each parameter, all possible interactions between the selected predictors were tested and left in the model if the *P* value was less than 0.05. No interaction term was tested for parameter ρ because it already represented a three-way interaction term. This procedure was repeated on 100 bootstrap samples of size 1027. A variable or interaction term was kept in the final model if it was selected in more than half of the 100 bootstrap samples.

### Model performance

The model performance was assessed by discriminative accuracy and risk calibration, both within the derivation cohort and validation cohort. The discriminative accuracy was examined by calculating the area under the receiver operating characteristic curve (AUC). In the derivation cohort, a bootstrap cross-validation procedure was applied, in which the AUC statistics were calculated from the predictions performed on a random sample extracted with a replacement of size 1027. This process was repeated 1000 times, and the AUC statistic was calculated for each of these bootstrap samples. The model performance was assessed by calculating the AUC and 95% CI at 3 and 5 years after surgery and by plotting AUC changes over time. We also calculated the difference between the AUC derived from the validation sample and the external validation sample. We first randomly selected with replacement 200 samples from the internal samples and 200 samples from the external sample. In each bootstrap selection, we calculated the AUC at 3 and 5 years after surgery both for all-cause and disease-specific mortality. We then computed the 95% CI of the difference of the AUC values, as the 2.5–97.5% quantile intervals over the 200 bootstrapped AUC difference. To assess the risk calibration for the internal and external validation, Hosmer–Lemeshow calibration plots are reported showing the level of agreement between the predicted and observed proportions of deaths across tenths of predicted risks. The goodness of fit model was examined by the Hosmer–Lemeshow test, with the null hypothesis being that the observed and expected proportions were the same.

### Interactive calculator of survival probability

An interactive calculator was created to compute the probability of all-cause and disease-specific mortality at time t given that the patient has survived until time *t*_0_ after surgery (where *t*_0_ is greater or equal to one postsurgery year), and depending on the individual patient’s set of predictors. The calculator is available at: https://sites.google.com/view/pcsec/home.

The cumulative incidence functions for the competing risk esophageal cancer is ${\text{CIF}}_{\text{cancer}}(t)=[1-S_{4}(t|d_{i}=1,x_{i}^{T};\beta^{T})]f_{2}(d_{i}=1|x_{i}^{T};\alpha^{T})$ , the cumulative incidence function for competing risk from another cause of death is ${\text{CIF}}_{\text{other}}(t)=[1-S_{4}(t|d_{i}=2,x_{i}^{T};\beta^{T})][1-f_{2}(d_{i}=1|x_{i}^{T};\alpha )]$ , and the total cumulative incidence function is $\text{CIF}(t)={\text{CIF}}_{\text{cancer}}(t)+{\text{CIF}}_{\text{other}}(t)$ .

For a patient who has survived until *t*_0_, where *t*_0_ > 1, the conditional CIF at time t for competing risk esophageal cancer is: ${\text{CIF}}_{\text{cancer}}\left(t|t>t0\right)=\left[{\mathrm{CIF}}_{\mathrm{cancer}}\left(t+t0\right)-{\mathrm{CIF}}_{\mathrm{cancer}}\left(t0\right)\right]/[1-\text{CIF}(t0)]$ , while the conditional all-cause mortality CIF was CIF(t|t>t0)=1−[1−CIF(t+t0)]/[1−CIF(t0)] .

All statistical analyses followed a predefined protocol and were performed by two senior statisticians (G.S. and M.B.) using command *mlexp* in the statistical software Stata (Release 15; StataCorp.) to maximize the likelihood function.

## Results

### Patients

Characteristics of the study participants are shown in Table 1. The derivation cohort included 1027 patients, and the majority were men (*n*=758, 73.8%). The validation cohort included 558 patients (444 men, 79.6%). The medians of age at surgery were 65.1 [interquartile range (IQR): 58.2–71.6) years in the derivation cohort and 66.7 (IQR: 59.8–71.7) years in the validation cohort. Most patients had pathological tumor stages 0–I or II in both cohorts (71.1% in the derivation cohort and 58.5% in the validation cohort). The median follow-up was 2.5 (IQR: 0.7–4.0) years in the derivation cohort and 4.0 (IQR: 1.1–4.0) years in the validation cohort. Death within 5 years of surgery occurred in 588 (57.2%) of patients in the derivation cohort and 268 (48.0%) in the validation cohort, and most died from esophageal cancer [516 (89.3%) in the derivation cohort and 234 (87.3%) in the validation cohort]. Baseline characteristics were statistically different (Table 1).

**Table 1:** Sociodemographic and clinical characteristics of two cohorts of 1-year survivors after surgery for esophageal cancer.

	Derivation cohort	Validation cohort	
Characteristics	*N* (%)	*N* (%)	*P* value*
Total	1027 (100)	558 (100)	
Age [median (interquartile range)]	65.1 (58.2–71.6)	66.7 (59.8–71.7)	0.008
Sex			0.010
Men	758 (73.8)	444 (79.6)	
Women	269 (26.2)	114 (20.4)	
Education (years)			<0.001
≤9	491 (47.8)	180 (32.6)	
10–12	383 (27.3)	251 (45.5)	
>12	153 (14.9)	121 (21.9)	
Charlson comorbidity index			<0.001
0	575 (56.0)	220 (39.4)	
1	436 (29.3)	196 (35.1)	
≥2	151 (14.7)	142 (24.5)	
Tumor histology			<0.001
Adenocarcinoma	494 (48.1)	452 (81.0)	
Squamous cell carcinoma	533 (51.9)	106 (19.0)	
Chemo(radio)therapy	341 (33.2)	387 (69.4)	<0.001
Pathological tumor stage			<0.001
0–I	331 (32.2)	235 (42.7)	
II	399 (38.9)	87 (15.8)	
III	254 (24.7)	169 (30.7)	
IV	43 (4.2)	59 (10.7)	
Resection margin status			<0.001
Radical (R0)	933 (90.9)	455 (82.7)	
Nonradical (R1 or R2)	94 (9.2)	95 (17.3)	
Reoperation within 30 days	86 (8.4)	28 (5.0)	0.014
Five-year all-cause mortality	588 (57.2)	268 (48.0)	<0.001
Five-year disease-specific mortality	516 (50.2)	234 (41.9)	0.002

* Pearson’s *χ* ^2^ for categorical variables; quantile regression for continuous variables.

### Final model

For each parameter, the frequency of each candidate predictor kept in the model and the number and percentage of times *P* greater than or equal to 0.05 during the variable selection procedure are shown in Supplementary Table 1, Supplemental Digital Content 2, http://links.lww.com/JS9/A225 and Table 2, Supplemental Digital Content 2, http://links.lww.com/JS9/A225. The coefficient and 95% CI for each predictor in the final model are shown in Table 2. The final model included those predictors that satisfied the condition described in section ‘Model development’ above, that is, five predictors for function $f_{2}$ : age, tumor histology, chemo(radio)therapy, pathological tumor stage, and resection margin status; and seven predictors for parameter *η* in function S_4_: age, sex, education, tumor histology, pathological tumor stage, resection margin status, and reoperation. Charlson comorbidity was excluded from the final model because the *P* value was greater than 0.05 in greater than 50 bootstrap samples.

**Table 2:** Coefficients (log odds) and 95% CI from prediction models of survival for up to 5 years among 1-year survivors of surgery for esophageal cancer.

Predictor	Parameter *α*	Parameter *η*	Parameter *ϕ*, *γ*, or *ρ*
Age	0.28 (0.08; 0.49)	−0.15 (−0.30; −0.00)	
Sex			
Male		Reference	
Female		0.29 (−0.1; 0.60)	
Education (years)			
≤12		Reference	
>12		0.38 (0.00; 0.77)	
Tumor histology			
Adenocarcinoma	Reference	Reference	
Squamous cell carcinoma	0.58 (0.20; 0.96)	−0.26 (−0.53; 0.02)	
Chemo(radio)therapy			
No	Reference		
Yes	0.45 (0.03; 0.87)		
Pathological tumor stage			
0–I	Reference	Referencea	
II	1.57 (1.14; 2.00)	Referencea	
III	2.25 (1.69; 2.80)	−0.56 (−0.86; −0.26)	
IV	2.37 (1.23; 3.50)	−0.98 (−1.56; −0.40)	
Resection margin status			
Radical (R0)	Reference	Reference	
Nonradical (R1 or R2)	1.62 (0.50; 2.75)	−0.62 (−1.03; −0.20)	
Reoperation within 30 days			
No		Reference	
Yes		−0.46 (−0.89; −0.03)	
Constant	−1.26 (−1.67; −0.85)	2.67 (2.30; 3.04)	
Constant *ϕ*			−0.76 (−0.92; −0.59)
Constant *γ*			−2.20 (−2.65; −1.74)
Constant *ρ*			−0.42 (−0.64; −0.23)

a In this model, stage II is combined with stage 0–I.

**Figure 1:**
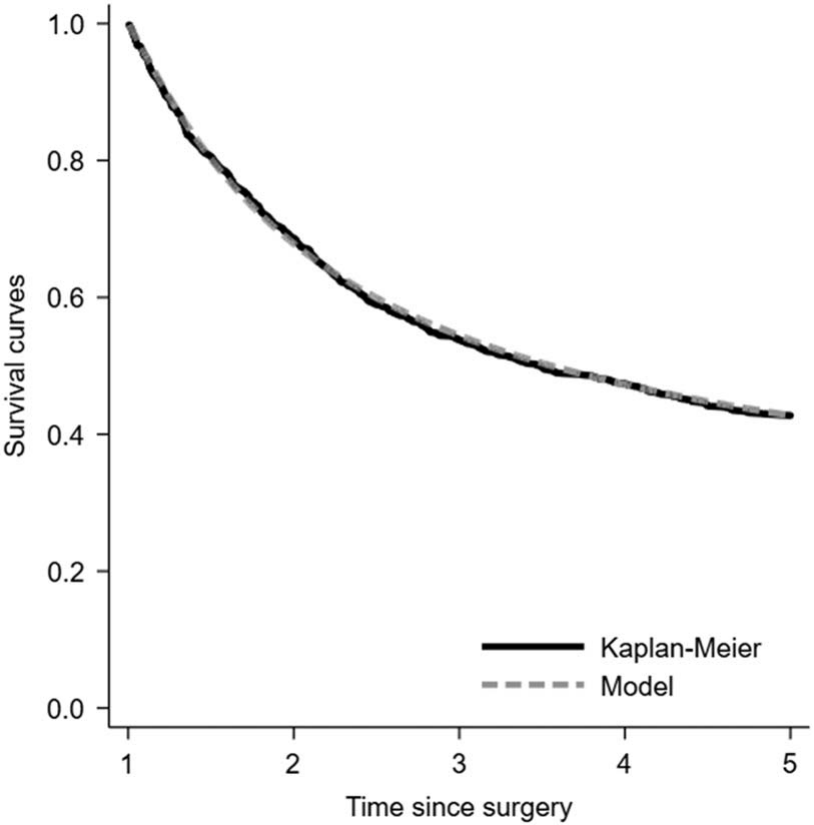
Goodness of fit comparing parametric estimates (Kaplan–Meier) and survival function *f*5 derived from a prediction model of survival for up to 5 years among 1-year survivors of surgery for esophageal cancer.

Figure 1 shows the goodness of fit of the final model by comparing the parametric estimate of the survival function (Kaplan–Meier curve) with the survival function $f_{5}$ estimates. The two curves overlapped, indicating that the model was correctly specified.

### Model performance

**Figure 2:**
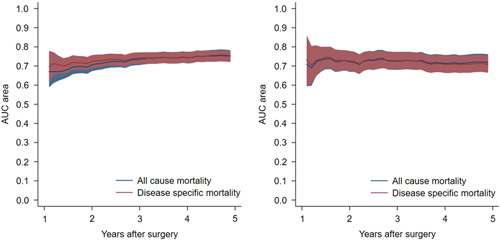
Area under the receiver operating characteristic curve (AUC) at different time points with internal validation (left) and external validation (right) for prediction models of survival for up to 5 years among 1-year survivors of surgery for esophageal cancer.

**Figure 3:**
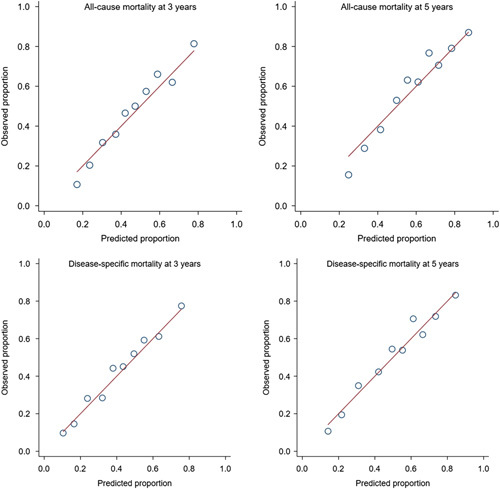
Hosmer–Lemeshow calibration plots between the predicted risks of death and observed proportions of deaths across tenths of predicted risk in internal validation of a prediction model of survival for up to 5 years among 1-year survivors of surgery for esophageal cancer.

**Figure 4:**
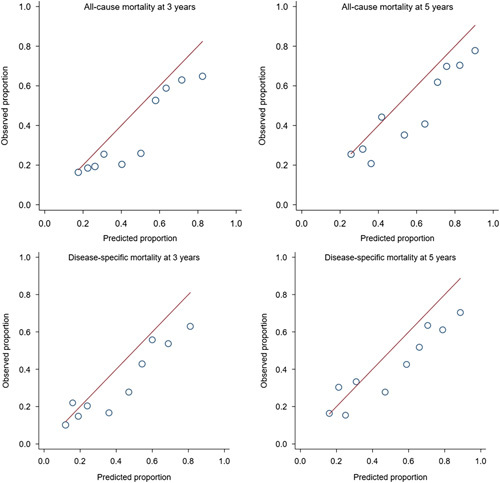
Hosmer–Lemeshow calibration plots between the predicted risks of death and observed proportions of deaths across tenths of predicted risk in external validation of prediction model of survival for up to 5 years among 1-year survivors of surgery for esophageal cancer.

The time-dependent AUC curves for all-cause and disease-specific mortality in internal cross-validation and external validation are shown in Figure 2. Among patients who survived 1 year after surgery, the derivation cohort showed medians of AUC of 0.74 (95% CI: 0.69–0.78) for 3-year all-cause mortality, 0.76 (95% CI: 0.72–0.79) for 5-year all-cause mortality, 0.74 (95% CI: 0.70–0.78) for 3-year disease-specific mortality, and 0.75 (95% CI: 0.72–0.79) for 5-year disease-specific mortality (Table 3). In the external validation cohort, the corresponding medians of AUC ranged from 0.71 to 0.73 (Table 3). There was no statistically significant difference between the AUC values derived for the external and internal validation samples (Table 3). The predicted and observed risks of mortality showed good agreement in both internal and external validation (Figs 3 and 4; *P*>0.05 in Hosmer–Lemeshow tests).

**Table 3:** Area under the receiver operating characteristic curve of the developed prediction models and their difference for 3-year and 5-year all-cause and disease-specific mortality presented as median (95% CI).

Outcomes	Internal validation	External validation	Difference
All-cause mortality			
Three years	0.74 (0.69; 0.78)	0.73 (0.68; 0.77)	0.01 (−0.04; 0.05)
Five years	0.76 (0.72; 0.79)	0.72 (0.67; 0.76)	0.04 (−0.01; 0.08)
Disease-specific mortality			
Three years	0.74 (0.70; 0.78)	0.73 (0.68; 0.77)	0.01 (−0.04; 0.06)
Five years	0.75 (0.72; 0.79)	0.71 (0.66; 0.75)	0.04 (−0.01; 0.10)

### Web-based calculator

To provide complete results for the prediction model for each patient, we developed an interactive web-tool to calculate all-cause and disease-specific mortality at any date *t* (*t*<5 years) in patients who had survived until any date t0 (1 years ≤*t*_0_<*t*) after esophageal cancer surgery. The web-tool is referred to above.

To illustrate how the web-tool can be used, let us, as an example, consider a 65-year-old male patient with 9 years of education, pathological tumor stage I, squamous cell carcinoma, no chemo(radio)therapy, tumor-free resection margins (R0), and no reoperation. Supplementary Figure 1a, Supplemental Digital Content 2, http://links.lww.com/JS9/A225 estimates that if he survived 1 year after surgery, his probability of death within the following 2 years (i.e. within 3 years of surgery) from any cause is 30.4% and from esophageal cancer is 21.7%. For the same patient having survived 2.6 years after surgery, Supplementary Figure 1b, Supplemental Digital Content 2, http://links.lww.com/JS9/A225 estimates a 5.8% probability of dying due to any cause and a 3.7% probability of death from esophageal cancer within 6 months.

## DISCUSSION

This study used two population-based cohorts to develop and validate a prediction model for projecting patients’ conditional survival after esophageal cancer surgery. The final predictors were age, sex, education, tumor histology, chemo(radio)therapy, tumor stage, resection margin status, and reoperation. The model showed good performance.

Among methodological strengths are the population-based cohort design with nearly complete inclusion, complete follow-up, accurate data on the exposure, outcomes, and predictors. We developed a novel biostatistical approach by creating a probability equation specifically for this study. The performance of the developed prediction model was assessed with both internal cross-validation and external validation in an independent cohort, which counteracted over-fitting. However, due to potential differences in patients’ characteristics, healthcare systems, and treatment across populations, the model remains to be validated in other countries. Another limitation was the lack of data on some potential predictors, that is, anthropometric measures and lifestyle factors.

We addressed the research question posed by a patient (‘What is my specific and individual probability of surviving given that I now underwent surgery some time ago’) in mathematical terms and expressed by the likelihood function. Several functional forms for the survival function were tested to determine which best fitted the data. The prediction capability of the patient’s characteristics was verified for all parameters of the likelihood function while including competing risks and interval censoring in the model. Although the likelihood function was complex, the program only required a few lines of code and was computationally fast. The explicit formulation of the likelihood function facilitated the postprocessing procedure and particularly the derivation of the cumulative incidence function and the AUC curves. However, writing of the likelihood function required deep understanding of the underlying clinical setting and statistical methods. Because the number of predictor variables was limited, the likelihood function did not need regularization^24^. This may limit its direct use in settings where several predictors (big data) are present.

A handful of models have been developed for projecting conditional postoperative survival in other cancers, including gastric cancer and penile cancer^25^,^26^. Because of the prognostic factors differ considerably between cancer types, we these models are not applicable to esophageal cancer patients. Some models have been developed for predicting long-term survival in esophageal cancer patients at baseline^13^–^17^, including one from our group^17^. By combining information on various prognostic factors, these models from previous studies have shown moderate to good performance, with AUC values ranging from 0.6 to 0.8. If conditional survival was estimated, this was limited to the probability of survival for certain additional years (e.g. 3 years) given integer numbers of years of accumulated survival. In the present study, we instead created a probability equation, which provides a truly ‘dynamic’ estimation of postoperative survival.

The present study is the first to estimate the conditional survival in patients with esophageal cancer, that is, how the probability of survival changes (improves) continuously for each day after surgery. This knowledge should be of great relevance for patients and healthcare. Individual patients’ prognosis at any given date after surgery is better provided by a valid conditional prediction model that takes the postsurgery survival time into account. This may improve healthcare by making the clinical follow-up more tailored. The online model is easy to use and may thus be a valuable tool for clinicians when they follow-up their patients after surgery. Patients often ask about their chance of survival. Rather than using baseline data, the online tool data would be more accurate for each individual patient. The chance of survival increases over time after surgery, so this information should not be alarming for most patients.

In conclusion, this study using two independent population-based cohorts provides a new model for individualized estimation of survival in patients who have undergone curative surgery for esophageal cancer, conditional on the time they have survived thus far. The developed model showed good performance in both internal and external validation, and may help making postoperative healthcare and follow-up more individualized.

## Ethical approval

The study was approved by the Regional Ethical Review Board in Stockholm (2107/141-31/2).

## Sources of funding

The work was supported by the Swedish Research Council (2019-00209); Swedish Cancer Society (21 1489); Stockholm Cancer Society (201163); Ihres Foundation (961440); and Julin Foundation. The funding sources had no role in the study.

## Author contributions

S.X., G.S., P.L., and J.L.: conceptualization. G.S.: data curation. G.S. and M.B.: formal analysis. S.X. and J.L.: funding acquisition. G.S. and M.B.: methodology. G.S. and M.B.: software. P.L. and J.L.: supervision. G.S. and M.B.: validation. S.X. and G.S.: writing – original draft. E.G., P.L. and J.L.: writing – review and editing.

## Conflicts of interest disclosure

None.

## Research registration unique identifying number (UIN)

1. Name of the registry: Clinicaltrials.gov
2. Unique identifying number or registration ID: NCT05540119
3. Hyperlink to your specific registration (must be publicly accessible and will be checked): www.clinicaltrials.gov/ct2/show/NCT05540119

## Guarantor

Professor Jesper Lagergren, MD, PhD, Upper Gastrointestinal Surgery, Department of Molecular Medicine and Surgery, Karolinska Institutet, Retzius Street 13a, 4th Floor, Stockholm 17177, Sweden. E-mail: jesper.lagergren@ki.se

## Data statement

Data may be shared on request to the corresponding author, but will require permissions of the Ethical Review Board and the governmental authorities that contributed with data used in this article, that is, the Swedish National Board of Health and Welfare and Statistics Sweden.

## Provenance and peer review

Not commissioned, externally peer-reviewed.

## Acknowledgments

The authors thank Dr Lars Arnberg for his valuable contribution to the idea of this study and valuable input on the manuscript from a patient perspective.

## Supplementary Material
