# Supplementary material for: Prediction of conditional survival in esophageal cancer in a population-based cohort study
Source: Int J Surg. 2023 Apr 3;109(5):1141–8. doi: 10.1097/JS9.0000000000000347 (PMC10389626; doi:10.1097/JS9.0000000000000347)
Supplement: Supplementary file 2 [file js9-109-1141-s002.docx]

**Supplementary Table 1.** Variable selection for parameters α and η based on the 100 random samples with replacement. Frequency of variables has been kept in the final model and the number and percentages of time p≥0.05.

| **Variables** | **Parameter α** | | | **Parameter η** | | |
| --- | --- | --- | --- | --- | --- | --- |
|  | **N** | **P≥0.05** | **%rejected** | **N** | **P≥0.05** | **%rejected** |
| Age | 100 | 51 | 51 | 100 | 35 | 35 |
| Resection margin status | 99 | 11 | 11 | 100 | 44 | 44 |
| Tumor stage II | 83 | 2 | 2 | 14 | 2 | 14 |
| Tumor stage III | 100 | 3 | 3 | 100 | 3 | 3 |
| Tumor stage IV | 88 | 3 | 3 | 96 | 8 | 8 |
| Tumor histology | 89 | 7 | 8 | 86 | 25 | 29 |
| Reoperation within 30 days | 31 | 2 | 7 | 61 | 44 | 72 |
| Neoadjuvant therapy | 50 | 3 | 6 | 12 | 3 | 25 |
| Sex | 0 |  |  | 100 | 82 | 82 |
| Education ≤12 years | 5 | 1 | 20 | 3 | 0 | 0 |
| Education >12 years | 11 | 4 | 36 | 63 | 20 | 32 |
| Charlson index = 1 | 18 | 2 | 11 | 5 | 1 | 20 |
| Charlson index ≥2 | 12 | 4 | 33 | 12 | 2 | 17 |
| Age interaction with: |  |  |  |  |  |  |
| Resection margin status | 5 | 0 | 0 | 27 | 6 | 22 |
| Tumor stage II | 1 | 0 | 0 | 2 | 1 | 50 |
| Tumor stage III | 2 | 0 | 0 | 11 | 0 | 0 |
| Tumor stage IV | 3 | 1 | 33 | 45 | 8 | 18 |
| Tumor histology | 9 | 2 | 22 | 21 | 3 | 14 |
| Reoperation within 30 days | 6 | 1 | 17 | 6 | 6 | 100 |
| Neoadjuvant therapy | 9 | 5 | 56 | 1 | 0 | 0 |
| Sex | 0 |  |  | 22 | 5 | 23 |
| Education >12 years | 3 | 2 | 67 | 2 | 1 | 50 |
| Charlson index = 1 | 2 | 0 |  | 0 |  |  |
| Charlson index ≥2 | 2 | 1 | 50 | 3 | 1 | 33 |
| Resection margin status interaction with: |  |  |  |  |  |  |
| Tumor stage II | 7 | 5 | 71 | 2 | 1 | 50 |
| Tumor stage III | 0 |  |  | 28 | 5 | 18 |
| Tumor stage IV | 0 |  |  | 12 | 0 | 0 |
| Tumor histology | 6 | 5 | 83 | 27 | 3 | 11 |
| Reoperation within 30 days | 9 | 0 | 0 | 43 | 2 | 5 |
| Neoadjuvant therapy | 1 | 1 | 100 | 2 | 0 | 0 |
| Education 9-12 years | 0 |  |  | 2 | 0 | 0 |
| Education >12 years | 0 |  |  | 4 | 1 | 25 |
| Charlson index ≥2 | 1 | 0 |  | 3 | 1 | 33 |
| Tumor stage II interaction with: |  |  |  |  |  |  |
| Tumor histology | 8 | 1 | 13 | 5 | 3 | 60 |
| Reoperation within 30 days | 0 |  |  | 1 | 1 | 100 |
| Neoadjuvant therapy | 4 | 2 | 50 | 1 | 0 | 0 |
| Education 9-12 years | 1 | 0 | 0 | 0 |  |  |
| Education >12 | 0 |  |  | 2 | 0 | 0 |
| Charlson index ≥2 | 1 | 0 | 0 | 0 |  |  |
| Tumor stage III interaction with: |  |  |  |  |  |  |
| Tumor histology | 19 | 2 | 11 | 33 | 3 | 9 |
| Reoperation within 30 days | 0 |  |  | 9 | 2 | 22 |
| Neoadjuvant therapy | 25 | 0 | 0 | 3 | 1 | 33 |
| Education 9-12 years | 1 | 0 | 0 | 0 |  |  |
| Education >12 years | 1 | 0 | 0 | 4 | 1 | 25 |
| Charlson index = 1 | 3 | 0 | 0 | 0 |  |  |
| Charlson index ≥2 | 0 |  |  | 5 | 1 | 20 |
| Tumor stage IV interaction with: |  |  |  |  |  |  |
| Tumor histology | 1 | 0 | 0 | 24 | 0 | 0 |
| Neoadjuvant therapy | 4 | 0 | 0 | 3 | 1 | 33 |
| Education >12 years | 0 |  |  | 10 | 1 | 10 |
| Charlson index = 1 | 0 |  |  | 2 | 0 | 0 |
| Charlson index ≥2 | 0 |  |  | 2 | 1 | 50 |
| Tumor histology interaction with: |  |  |  |  |  |  |
| Reoperation within 30 days | 1 | 0 | 0 | 10 | 1 | 10 |
| Neoadjuvant therapy | 19 | 0 | 0 | 0 |  |  |
| Education >12 years | 2 | 0 | 0 | 5 | 2 | 40 |
| Charlson index = 1 | 1 | 0 | 0 | 3 | 0 | 0 |
| Charlson index ≥2 | 2 | 0 | 0 | 2 | 0 | 0 |
| Reoperation within 30 days interaction with: |  |  |  |  |  |  |
| Neoadjuvant therapy | 3 | 0 | 0 | 2 | 0 | 0 |
| Education 9-12 years | 1 | 0 | 0 | 0 |  |  |
| Education >12 years | 0 |  |  | 9 | 1 | 11 |
| Charlson index ≥2 | 2 | 0 | 0 | 1 | 0 | 0 |
| Neoadjuvant therapy interaction with: |  |  |  |  |  |  |
| Education 9-12 years | 0 |  |  | 1 | 0 | 0 |
| Charlson index ≥2 | 0 |  |  | 1 | 0 | 0 |
| Sex interaction with: |  |  |  |  |  |  |
| Education >12 years | 0 |  |  | 6 | 2 | 33 |
| Charlson index ≥2 | 0 |  |  | 5 | 0 | 0 |
| Education 9-12 years interaction with: |  |  |  |  |  |  |
| Charlson index ≥2 | 1 | 0 | 0 | 0 |  |  |
| Education >12 years interaction with: |  |  |  |  |  |  |
| Charlson index = 1 | 0 |  |  | 1 | 0 | 0 |
| Charlson index ≥2 | 0 |  |  | 2 | 0 | 0 |

Only interactions that were present at least once in a model are reported.

**Supplementary Table 2.** Variable selection for parameters *φ*, *γ*, and *ρ* based on the 100 random samples with replacement. Frequency of variables was kept in the final model and the number and percentages of time p≥0.05.

| **Variables** | **Parameter *φ*** | | | **Parameter *γ*** | | | **Parameter *ρ*** | | |
| --- | --- | --- | --- | --- | --- | --- | --- | --- | --- |
|  | **N** | **P≥0.05** | **%rejected** | **N** | **P≥0.05** | **%rejected** | **N** | **P≥0.05** | **%rejected** |
| Resection margin status | 5 | 3 | 60 | 4 | 2 | 50 | 1 | 1 | 100 |
| Tumor stage II | 28 | 15 | 54 | 18 | 16 | 89 | 16 | 6 | 38 |
| Tumor stage III | 42 | 18 | 43 | 14 | 7 | 50 | 2 | 0 | 0 |
| Tumor stage IV | 18 | 1 | 5.6 | 10 | 1 | 10 | 3 | 1 | 33 |
| Tumor histology | 8 | 0 | 0 | 16 | 8 | 50 | 11 | 3 | 27 |
| Reoperation within 30 days | 8 | 0 | 0 | 7 | 0 | 0 | 3 | 2 | 67 |
| Neoadjuvant therapy | 19 | 9 | 47 | 3 | 1 | 33 | 11 | 4 | 36 |
| Education ≤12 years | 22 | 2 | 9.1 | 2 | 2 | 100 | 1 | 0 | 0 |
| Education >12 years | 27 | 9 | 33 | 5 | 1 | 20 | 2 | 0 | 0 |
| Charlson index = 1 | 24 | 6 | 25 | 4 | 3 | 75 | 4 | 1 | 25 |
| Charlson index ≥2 | 13 | 3 | 23 | 8 | 2 | 25 | 4 | 3 | 75 |
| Resection margin status interaction with: |  |  |  |  |  |  |  |  |  |
| Tumor stage II | 1 | 0 | 0 | 0 |  |  |  |  |  |
| Charlson index = 1 | 1 | 0 | 0 | 0 |  |  |  |  |  |
| Tumor stage II interaction with: |  |  |  |  |  |  |  |  |  |
| Reoperation within 30 days | 1 | 0 | 0 | 0 |  |  |  |  |  |
| Education 9-12 years | 2 | 0 | 0 | 1 | 0 | 0 |  |  |  |
| Education >12 years | 1 | 0 | 0 | 0 |  |  |  |  |  |
| Tumor stage III interaction with: |  |  |  |  |  |  |  |  |  |
| Reoperation within 30 days | 2 | 0 | 0 | 0 |  |  |  |  |  |
| Neoadjuvant therapy | 1 | 0 | 0 | 0 |  |  |  |  |  |
| Education 9-12 years | 1 | 0 | 0 | 0 |  |  |  |  |  |
| Charlson index ≥2 | 2 | 0 | 0 | 0 |  |  |  |  |  |
| Tumor stage IV interaction with: |  |  |  |  |  |  |  |  |  |
| Education >12 years | 1 | 0 | 0 | 0 |  |  |  |  |  |
| Charlson index = 1 | 1 | 0 | 0 | 0 |  |  |  |  |  |
| Charlson index ≥2 | 1 | 0 | 0 | 0 |  |  |  |  |  |
| Tumor histology interaction with: |  |  |  |  |  |  |  |  |  |
| Charlson index = 1 | 1 | 0 | 0 | 1 | 0 | 0 |  |  |  |
| Charlson index ≥2 | 0 |  |  | 2 | 0 | 0 |  |  |  |
| Neoadjuvant therapy interaction with: |  |  |  |  |  |  |  |  |  |
| Education >12 years | 1 | 0 | 0 | 0 |  |  |  |  |  |
| Charlson index = 1 | 1 | 0 | 0 | 0 |  |  |  |  |  |
| Charlson index ≥2 | 1 | 0 | 0 | 0 |  |  |  |  |  |
| Education >12 years interaction with: |  |  |  |  |  |  |  |  |  |
| Charlson index = 1 | 1 | 0 | 0 | 0 |  |  |  |  |  |

Only interactions present at least once in a model are reported.

a)
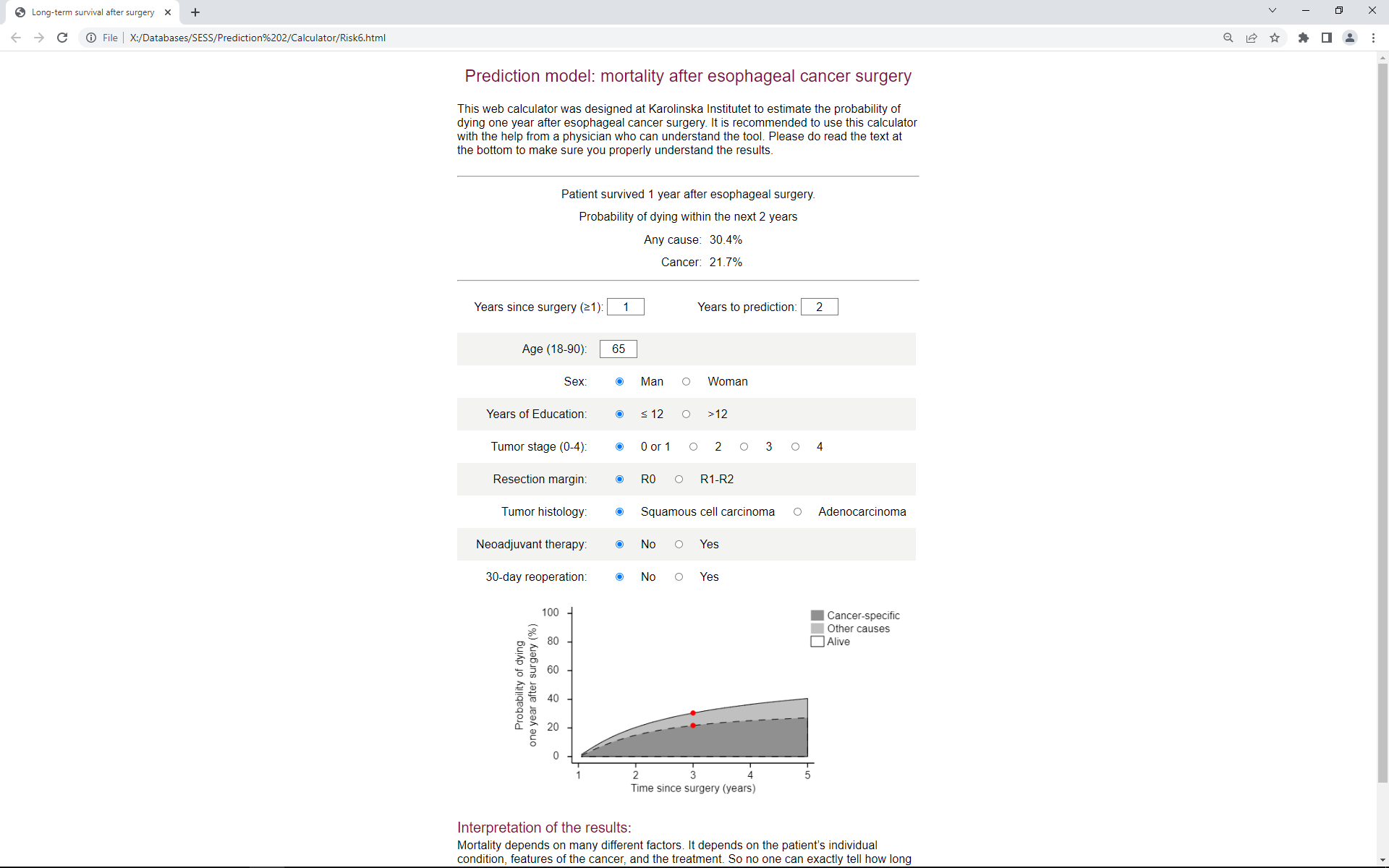
 b)
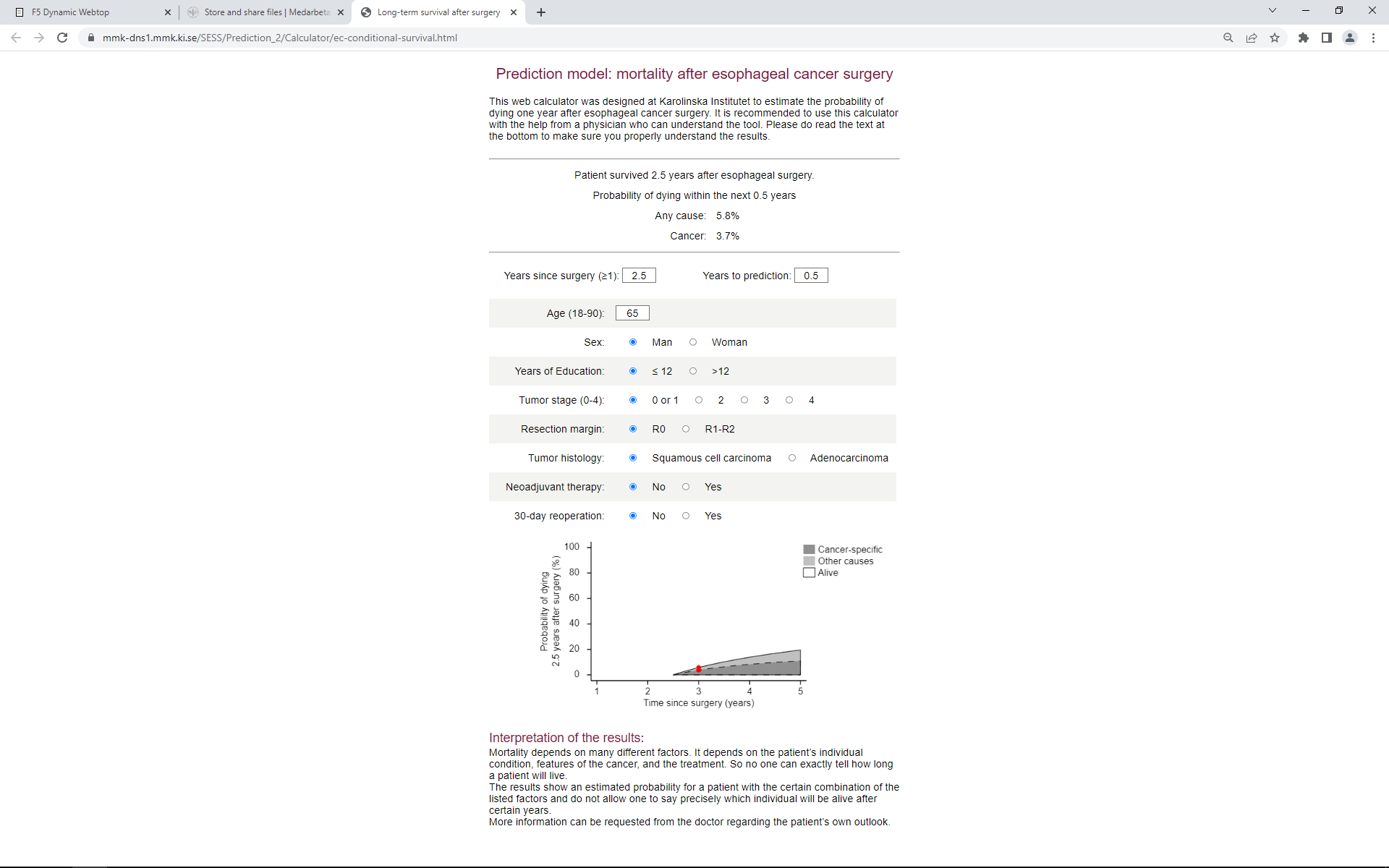


**Supplementary Figure 1.** Screenshot of an interactive tool for predicting conditional survival among 1-year survivors of surgery for esophageal canc

a) The graph reports predictions for a patient who has survived the first year after surgery; the red dots and the reported percentages are the probability of dying within two years of any cause (30.4%) or esophageal cancer (21.7%).

b) The graph reports predictions for a patient who has survived two and a half years after surgery; the red dots and the reported percentages are the probability within the next 6 months of dying of any cause (5.8%) or esophageal cancer (3.7%).
